# Words, Words, Words

**Published:** 1900-02

**Authors:** 


### "WORDS, WORDS, WORDS."

It is not unusual to note in medical journals a tendency on the part of writers to clothe their ideas, or to cloak their lack of them, in Johnsonese English. It is like walking on stilts when the feet will serve equally well. As an example, we quote the following, which, for limpid clearness, reminds one of the definition of osteopathy as furnished by the osteopaths: "If we want to effectuate the transformation of a deflected position into a flected position, the skull has to pass through a transient position in which the fetal axis is elongated; to this effect manipulations applied to the skull only are not sufficient. The main point is to dislocate the fetal trunk upward and sideward." Why not say change instead of "effectuate the transformation"? This would add clearness and lend some dignity to a very lame sentence. There are some writers of note in the medical world who are the most wanton offenders in this regard. The crisp, concise "sacrificial" style of the present has not reached their archaic observation, and they continue to employ the "*sesquipedalian* verbiage" which was current early in the century under the false notion that such is the language of science. Compare the terseness, strength and vigor of such masters of science and language as Huxley, Darwin and Tyndall with their emasculate flabbiness, and observe how crystal is the idea in the one instance and how murky and turbid in the other. This tractile method of expression is frequently true of the fact to be expressed, which becomes drawn out to such length and thinness as almost to lose its continuity. It is well to remember the remark of Alex. Stephens to a young literary aspirant, that the goose is the only bird that lays more eggs than it can cover.

W.

---

THE name of George Villiers, Duke of Buckingham, is that of one of the most brilliant, versatile and profligate characters in English history. Heir to the greatest name and the greatest estate at the time in England, the boyhood companion and sharer in the vicissitudes of that monarch "who never said a foolish thing and never did a wise one," Villiers was at once the beau-ideal of the gallant and the wit and the type of the political intriguer. The most notedly licentious of a licentious age, a bully and braggart, living in open adultery, his life comprised the rôles of *le jeun homme pauvre,* the mountebank, astrologer, the poet, courtier, theological writer. Everything he undertook was marked by dash, brilliancy and irresoluteness. The finest gentleman in all England, according to Louis XIV. the only one, after tasting all the pleasures of life and long familiar with all its vices, died poor in fortune, broken in mind and body, at the age of sixty-one, April 16, 1687. His faithful biographer, Brian Fairfax, gives the fullest and most trustworthy account of the circumstances surrounding his last moments and the cause of his death. The latter, viewed from the present standpoint of our knowledge, is a matter of medical interest as bearing upon the history of a complaint that greatly engages the attention of the profession. Tired and overheated after a fox-hunt, the Duke of Buckingham threw himself down in the wet grass to cool. Not many hours after he was taken with a violent pain in the abdomen, accompanied by the usual symptoms of shock, coldness and prostration. This continued for twelve hours, after which the pain left him and he expressed himself as feeling so well that he was sure there was not much the matter with him inside. This improvement was deceitful, for he rapidly began to sink and died. The diagnosis in the nomenclature of the period was "inflammation of the bowels, ending in mortification." In spite of the number of details lacking, the account of his illness and death points very strongly to fulminating appendicitis ending in gangrene. The rapid onset after sudden cooling of the body, the excessive pain referred to the abdomen, the illusory betterment in the symptoms preceding death, at once suggest the clinical course and termination without surgical intervention of a rapidly fatal case of appendicitis.

It is more than likely that a study of history from a medical viewpoint would develop the fact that many diseases now regarded as products of modern civilization are rather products of modern knowledge, and are really as ancient as the fly in the amber. W.

